# The Relevance of CD117-Immunocytochemistry Staining Patterns to Mutational Exon-11 in *c-kit* Detected by PCR from Fine-Needle Aspirated Canine Mast Cell Tumor Cells

**DOI:** 10.1155/2014/787498

**Published:** 2014-02-18

**Authors:** A. Sailasuta, D. Ketpun, P. Piyaviriyakul, S. Theerawatanasirikul, P. Theewasutrakul, A. Rungsipipat

**Affiliations:** ^1^STAR: Molecular Biology Research on Animal Oncology, Department of Pathology, Faculty of Veterinary Science, Chulalongkorn University, Bangkok 10330, Thailand; ^2^Veterinary Oncology Unit, Small Animal Teaching Hospital, Faculty of Veterinary Science, Chulalongkorn University, Bangkok 10330, Thailand; ^3^Biochemistry Division, Department of Physiology, Faculty of Veterinary Science, Chulalongkorn University, Bangkok 10330, Thailand; ^4^Department of Anatomy, Faculty of Veterinary Medicine, Kasetsart University, Bangkok 10900, Thailand

## Abstract

Canine cutaneous mast cell tumors (MCT) are the lethal skin tumors. The biological behavior of the MCT cells is quite varied and unpredictable. Almost MCT dogs usually require a rapid diagnosis and therapy. However, MCT diagnosis and prognosis are still dependent on histopathology which is rather inconvenient, time-consuming, painful, and harmful for some cases. Indeed, MCT can be easily accessible using fine-needle aspiration (FNA). In this study, our biopsy specimens were classified as low- and high-grade MCT based on the novel 2-tier histopathologic grading system. We have demonstrated the usage of fine-needle aspirated MCT cells (FNA-MCT cells) from these specimens as a primary cell source to study the distribution of CD117-immunocytochemistry (CD117-ICC) staining patterns and the frequency of internal tandem duplication- (ITD-) mutant exon-11 of *c-kit*. The result has substantially shown that there were three staining patterns identified in the cells. Only paranuclear pattern was significantly increased in the cells from high-grade MCT. Altogether, the ITD-mutant exon-11 was also detectable only in these cells. Therefore, the result has supported our hypothesis that there was an increased opportunity to observe a higher CD117-ICC staining pattern and exon-11 mutation in high-grade MCT; even these two parameters may not precisely indicate a histopathological grade.

## 1. Introduction

Canine cutaneous mast cell tumors (MCT) are the second-most prevalent life-threatening skin tumor of dogs. MCT usually give rise to a fatal paraneoplastic syndrome to affected dogs. For example, an excessive degranulation of various proinflammatory cytokines especially histamine and serotonin causes severe gastric perforation and systemic hypotension, respectively [1–3]. Therefore, almost MCT dogs require a swift diagnosis to establish an aggressive therapy.

Conventionally, MCT diagnosis is currently based upon Patnaik's MCT histopathologic grading system. This method provides much more useful information on MCT diagnosis, grading, prognosis, and also therapeutic planning. Grounded on this system, MCT could be classified into grades I, II, or III depending on each corresponding histopathologic morphology [4]. In hitherto, a novel MCT-histopathologic grading system, however, has been introduced into the veterinary pathology field. This 2-tier histopathologic grading classifies MCT into 2 groups: low-grade and high-grade MCT. This system has improved the concordance among veterinary pathologists on MCT grading and the relevance to survival time, when compared to the conventional Patnaik's grading system [5].

Naturally, the growth, maturation, and proliferation of mast cells are controlled by the function of a class-III receptor tyrosine kinase, called KIT (CD117). This protein is encoded by the protooncogene *c-kit*, which is predominantly expressed in many cells, such as Purkinje cells and interstitial cells of Cajal. For hematopoietic-originated cells, mast cells are the only cell type which abundantly expresses this protein. Moreover, this protooncogene is highly conserved among mammals. For example, canine *c-kit *is 88% and 82% homologous to human and mouse *c-kit*, respectively [6].

In 2004, Morini and colleagues used CD117-immunohistochemistry (CD117-IHC) to label KIT expressions in various formalin-fixed paraffin-embedded (FFPE) tissues of normal dogs and cats, including neoplastic specimens from both species as well. Intriguingly, normal skin mast cells of dogs showed strong positivity to CD117-IHC and there were at least two staining patterns seen in the study: membrane associated and diffuse cytoplasmic, respectively. Moreover, there was an additional CD117-staining pattern which was observed in the MCT specimens, the paranuclear pattern [7]. In addition, CD117-immunoexpression patterns, in particular the paranuclear, seemed to correspond to a histopathology grade, proliferation rate, and survival time, as described in a previously tissue-based study by Preziosi and his group. In that study, the investigators have shown that all MCT grade I had the diffuse pattern with or without the membranous pattern; meanwhile, all MCT grade III contained the paranuclear pattern. However, in the intermediate-grade MCT, all CD117-staining patterns were distributed in an equal frequency [8].

Later in 2007, Gil da Costa and coworkers also explained the correlation between the staining patterns of CD117-IHC to a histopathologic grade and proliferative rate in MCT. The study consequence strongly revealed that MCT samples containing the cytoplasmic staining pattern (diffuse or paranuclear) would increase the cell proliferation rate. Nevertheless, these two CD117-immunopositivities also related to higher histopathological grades grounded on Patnaik's grading system, within the same frequency [9].

Since CD117 plays a critical role in the regulation of growth, differentiation, and proliferation in normal mast cells, many investigators have tied its immunoexpression patterns to histopathologic grading, as a biparametric indicator used for MCT diagnosis. Although, the endeavor is still unsuccessful so far because of the variations in the results among different study groups. However, one consensus that might be valuable is that the paranuclear pattern seems to correspond to the intermediate or high-grade MCT, whose behavior of their neoplastic cells is commonly aggressive and unpredictable. Hence, the paranuclear pattern might be a good parameter which is used for predicting MCT biological behavior more precisely.

Albeit, the accurate tumorigenesis of MCT is still unknown up to date. However, mutations of *c-kit* as a driving force for MCT formation are recently evident, at least in part [3, 6, 10]. In particular, *internal tandem duplication* (ITD) of exon-11 causes a KIT malformation at the juxtamembrane domain, leading to an autophosphorylation without any specific ligand (*stem cell factor* (*SCF*)) binding. This abnormality results in an uncontrollable proliferation of mast cells and MCT formation. In general, almost mutations of *c-kit* are easily dissectible using polymerase chain reaction (PCR) [11, 12]. In case of ITD-mutant exon-11 of *c-kit*, the PCR product of mutant exon-11 usually consists of 3 subsidiary bands of 191 bp heterogenous normal alleles, 250 bp mutated alleles, and the heterodimerization (heteroduplex) between normal and mutant alleles as the largest fragments [13, 14]. ITD-mutant exon-11 has usually been observed increasingly in a higher-grade MCT. Moreover, in one previous study, the result has substantially shown that MCT patients possessing ITD-mutation in association with an aberrant KIT localization had the worse prognosis [14]. This might suggest that aberrant KIT expression might be a good indicator to predict an aggressive behavior of MCT, as well.

Even though, MCT diagnosis is in general valid by histopathology. However, the protocol may from time to time be impractical in some patients. In clinical practice, MCT diagnosis can be easily assessable using fine-needle aspiration (FNA) instead. This alternative diagnostic tool is usually painless, harmless, and inexpensive. In addition, FNA operation does not require any risky anesthetic procedures except only a suitable local anesthesia; therefore, vulnerable MCT dogs are not at risk. Recently, there have been evidences showing that the cytology of MCT cells collected by FNA greatly resembles, up to 92–96%, those diagnosed by histopathology, case by case [3]. Even though, the number of tumor cells obtained by FNA is lower than other methods, especially when compared to biopsy.

Taken together, according to the advantage of FNA over biopsy in some aspects especially its convenience and harmlessness plus with the cytologic mimicry of FNA cells to their tissue-based counterparts. Thus, it is reasonable to believe that MCT cells collected by FNA (FNA-MCT cells) should be a good representative for all MCT cells from each biopsy specimen. Moreover, they can indeed be exploited for studying MCT biology, which hopefully provides a deep information on MCT behavior involved in MCT diagnosis and prognosis. With our present knowledge, the information on these suppositions is still lacking, for example, the distribution of each CD117 staining pattern and the mutation frequency of mutant exon-11 in FNA-MCT cells.

Hence, in our recent study, the central questions were “Can FNA-MCT cells represent a good cell source for studying MCT biology behavior?” “Can we evaluate the distribution frequency of a CD117-immunolabeling pattern and mutant exon-11 using these cells?” and “Do these two parameters have any interconnection?” We hypothesized that FNA-MCT cells were the good representative for all MCT cells from tissue-based specimens graded by the novel 2-tier histopathologic grading system. We also speculated that these cells could be utilized to assess the distribution of CD117-staining patterns and mutation of exon-11 of *c-kit *and their preliminary interconnections. In addition, we proposed that a higher staining pattern and ITD mutation should be increased in the high-grade MCT.

Ultimately, the objectives of this study were (1) to demonstrate that FNA-MCT cells are the good representation for tissue-based MCT cells, (2) to use them for studying the distribution of CD117-immunolabeling patterns, (3) to assess the frequency of mutated exon-11 in *c-kit* in these cells, and (4) to preliminarily study the interconnection among these parameters before using them as a prognostic marker in the future.

## 2. Materials and Methods

### 2.1. Patient Selection and Specimen Collection

The dogs with various cutaneous masses were selected from the Veterinary Oncology Clinic, Small Animal Teaching Hospital, Faculty of Veterinary Science, Chulalongkorn University, Bangkok, Thailand, from 2010 to early 2012. Each dog came along with its corresponding client consent. Fine-needle aspiration was preliminarily performed for each patient to collect the FNA-MCT cells from the mass. The cells were stained by Giemsa dye and then investigated to rule out the MCT from other skin neoplasms. Finally, 30 MCT dogs confirmed by FNA were accepted to participate in the study with complete histories of physical examinations and therapeutic protocols. However, WHO 1980-clinical staging and TNM diagnostic criteria were excluded from this study.

Two sets of FNA-MCT cells from each case were collected. The first cell set was directly smeared on a silane-coated glass slide and prepared for CD117-immunocytochemistry (CD117-ICC). The cells in the second collection were resuspended in 250 *μ*L of PBS, which were used for investigating the ITD-mutant exon-11 in *c-kit*. Skin biopsy was also operated in the same case for novel 2-tier histopathology grading.

### 2.2. Histopathology

Each biopsied specimen was preserved in 10% buffered formalin for 5 days before tissue processing. The biopsied specimens were then submitted to the Pathology Unit, Department of Pathology, Faculty of Veterinary Science, Chulalongkorn University, for routine tissue-processing. The final histopathological diagnosis and grading were defined by three qualified veterinary pathologists using the novel 2-tier histopathologic grading system [5].

### 2.3. CD117-Immunocytochemistry (CD117-ICC)

Briefly, the FNA-MCT cells on the silane-coated glass slide were submerged in cold acetone for 5 minutes to preserve cell morphology immediately. Endogenous peroxidase was eliminated by 3% hydrogen peroxide (H_2_O_2_) at room temperature for 20 minutes. The nonspecific protein backgrounds were blocked by 1% bovine serum albumin (Sigma Aldrich, USA) at 37°C for 1 minute. Afterward, the neoplastic cells were incubated with rabbit-polyclonal anti-human *c-kit *antibodies (Dako, Denmark) as the primary antibody (1 : 600) for 30 minutes at room temperature and then washed by PBS. Further, the tumor cells were incubated with Envision peroxidase system (Dako, Denmark) for 30 minutes and washed again with PBS. The tumor cells were colored by reacting with the chromogenic substrate; 0.05% 3,3′-diaminobenzidine tetrahydrochloride (DAB) in 0.01 M Tris HCl at pH 7.6 combined with 0.03% H_2_O_2_, for 5 minutes, and then washed. Finally, the cells were counterstained using hematoxylin and visualized under the light microscope to assess CD117-ICC staining patterns in 10 HPF.

### 2.4. Genomic DNA Isolation

The genomic DNA of FNA-MCT cells from each case was purified and harvested using the commercial DNA isolation kit (Ultraclean Tissue & Cells DNA isolation kit, Mobio, USA; RI Technology, Thailand) with the standard protocol recommended by the manufacturer. Merely, the cells were homogenized by 500 *μ*L of TD1 solution provided in the isolation kit and mixed thoroughly with a vortex until the cells were dispersed. Cell lysate was loaded onto the silica spin-filter collecting tube and centrifuged at 10,000 ×G for 1 minute at room temperature, discarding the flow-through. The genomic DNA was bounded onto the silica spin-filter in this stage. Contaminants and enzymes were eradicated and inhibited by 400 *μ*L of TD2 solution. The genomic DNA-contained collecting tube was centrifuged at 10,000 ×G at room temperature for 1 minute and then discarded the flow-through. Afterward, the collecting tube was centrifuged again at 10,000 ×G at room temperature for 3 minutes to remove TD2 residues. The silica spin-filter was carefully transferred to a new clean 2 mL collecting tube to elute genomic DNA, by adding 50 *μ*L of TD3 solution (eluting buffer) and centrifuging at 10,000 ×G at room temperature for 1 minute. The genomic DNA was kept in TD3 solution at −20°C.

### 2.5. Polymerase Chain Reaction for Evaluation of Exon-11 Mutation in *c-kit*

The purified genomic DNA from each case was amplified using the modified PCR protocol recommended by School of Veterinary Medicine, Michigan State University, USA. The PCR cocktail consisted of 12.5 *μ*L of commercial PCR master mix (AcessQuick, Promega, USA), 5.5 *μ*L of distillated water, 3 *μ*L of genomic DNA template, and 2 *μ*L of forward primers and reverse primers each. The primers were designed from College of Veterinary Medicine, Michigan State University, USA, with the sequence of forward and reverse primers as *5*′*-CCA TGT ATG AAG TAC AGT GGA AG-3*′ of exon-11 and *5*′*-GTT CCC TAA AGT CAT TGT TAC ACG-3*′ of intron 11, respectively. The final PCR-reaction volume was 25 *μ*L. The PCR mixer was run in the thermocycler (G-Storm, USA) with the programmatically thermocyclic temperatures of 94°C for 4 minutes for initiated denaturation; 40 cycles of 94°C for 1 minute for denaturation, 55°C for 1 minute for DNA annealing, 72°C for 1 minute for DNA extension, and 72°C for 5 minutes for complete DNA extension. The amplified PCR product was kept at 4°C until analysis.

### 2.6. Agarose Gel Electrophoresis

The amplicons were dissipated using agarose-gel electrophoresis performing on 2% ethidium bromide-mixed agarose gel in 1x Tris-acetate-EDTA (TAE) solution at 100V for 40 minutes. Finally, the PCR products were visualized and analyzed using gel-documentation imaging system (Bio Rad, USA).

## 3. Results

### 3.1. Screening Cytology

**Figure 1:**
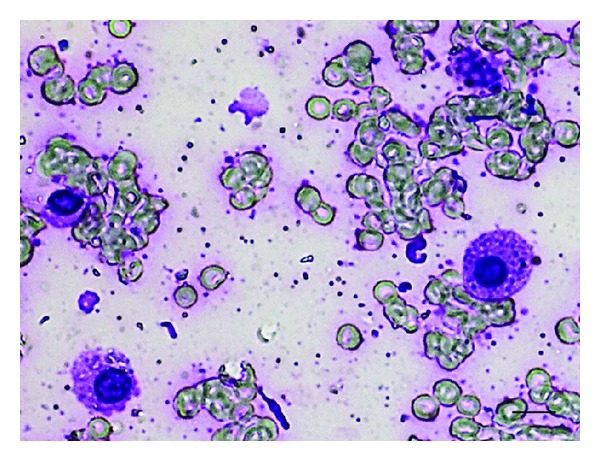
The morphology of MCT cells collected by FNA. The cells were round. The nuclei were deviated from concentric to eccentric. The cytoplasm was markedly basophilic filled by finely metachromatic granules. Notably, there were some cells containing fine vesicular granules in their cytoplasm (bar = 10 *μ*m).

The MCT cells in each aspirate were preliminary screened using Giemsa staining. The result consistently exhibited the persistence of MCT cells in each FNA sample. The cells were round with concentric or eccentric round nuclei. Their cytoplasm was basophilic fulfilled with fine metachromatic granules. The cells were approximately 10–15 *μ*m in size. Vesicular cytoplasm was also observed in some cells (Figure 1).

### 3.2. Histopathology

**Figure 2:**
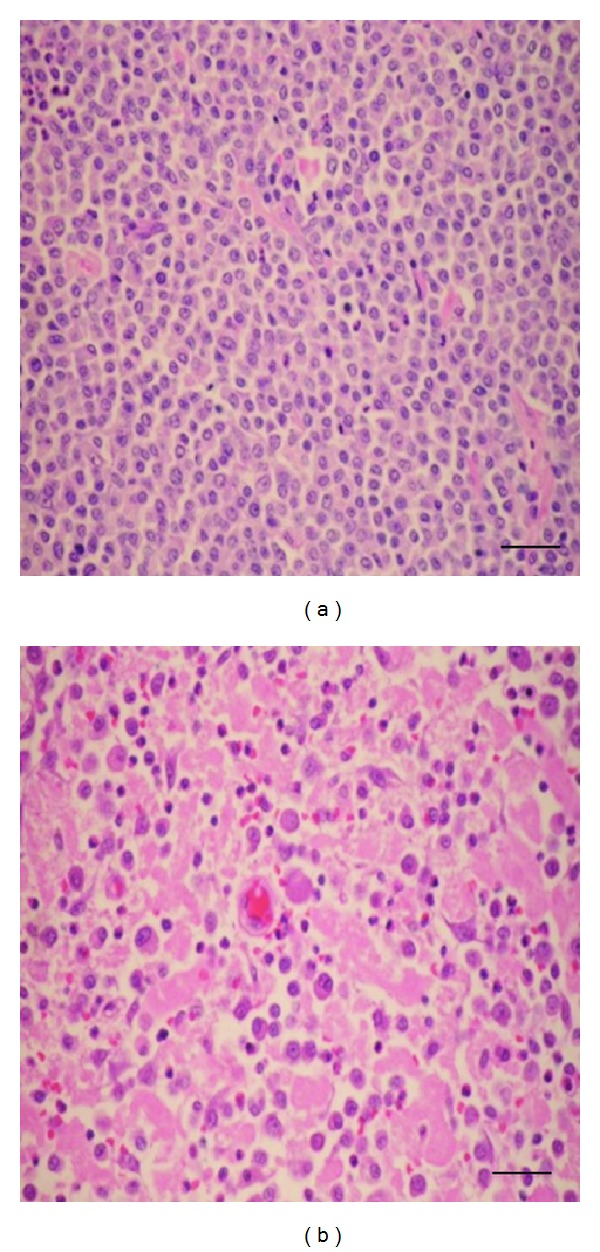
Two MCT histopathologic grades were classified according to the criteria of novel 2-tier histopathologic grading system [5]. (a) The low-grade MCT showed the uniformity of MCT cells in the section. The mitotic index was very low (<7 MI per 10 HPF). The multinucleated cells were rarely observed. (b) In high-grade MCT, the mitotic index was higher than 7 MI per HPF. Multinucleated cells with peculiar nuclei were commonly seen. Notably, necrotic tissues were obviously found in high-grade MCT (bar = 25 *μ*m).

Based on the novel 2-tier histopathologic grading, the MCT-biopsy specimens were classified into high grade and low grade in our study. There were 18 out of 30 specimens defined as low-grade MCT (60%); meanwhile, the others were high-grade MCT (12/30, 40%). The tissue section of low-grade MCT showed the uniformity in cell morphologies as seen in normal mast cells, such as well-differentiated cells, low mitotic index (<7 MI in 10 HPF), with less or without multinucleated neoplastic cells. The bizzare nuclei or 2-fold karyomegaly was absent in this study. In high-grade MCT, there were more mitotic figures (>7 MI in 10 HPF), multinucleated or odd-nuclei anaplastic cells. Moreover, multifocal necrosis was commonly observed in this grade (Figure 2). From the result in this study, there were 18 low-grade MCT and 12 high-grade MCT.

### 3.3. CD117-Immunocytochemistry Staining Patterns and Their Distributions

**Figure 3:**
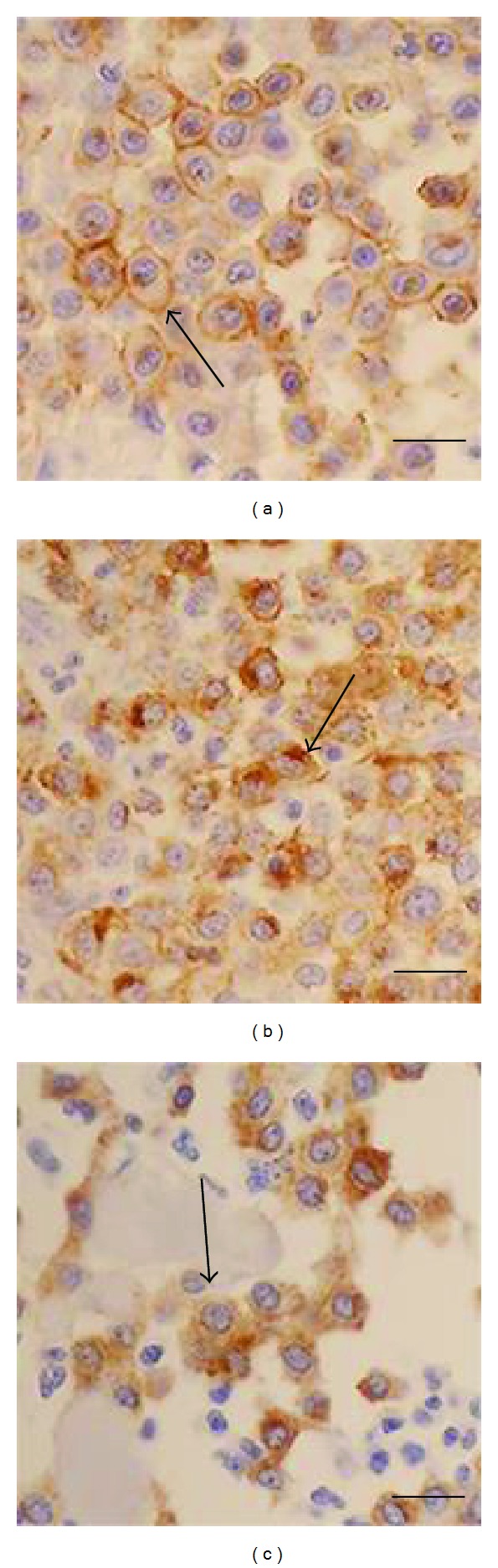
Three distinct CD117-immunocytochemistry staining patterns ((a) to (c)) have been presented in the study. (a) is the staining pattern 1 (perimembrane or membrane associated) which is highly distributed in low-grade MCT. (b) depicts the staining pattern 2 (paranuclear) and (c) exhibits the staining pattern 3 (diffuse), respectively. These last two staining patterns are almost present in high-grade MCT (bar = 10 *μ*m).

The staining patterns of CD117-ICC in FNA-MCT cells were substantially identical to those described in CD117-IHC as reported elsewhere: the membrane-associated, paranuclear, and diffuse patterns [9, 15]. The membrane-associated pattern (perimembrane, membranous, or pattern I) was strongly distributed on the plasma membranes in all low-grade MCT. The second pattern, paranuclear, was found in both low- and high-grade MCT. This pattern was characterized by cytoplasmic stipple adjacent to nuclear membranes. The third pattern was found abundantly in high-grade MCT. The immunolabeling always diffused throughout the cytoplasm of tumor cells. All CD117-ICC staining patterns were depicted in Figure 3. In this study, 16 out of 18 low-grade samples presented staining pattern I; meanwhile, the others (2/18) had staining pattern II. And, staining pattern III was undetectable in this grade. From 12 high-grade MCT specimens, 8 out of 12 cases contained CD117-ICC immunopositivity as the pattern II and 4 cases as the pattern III. Accordingly, a higher CD117-ICC staining pattern had a propensity to be observed in a higher MCT grade.

### 3.4. PCR Analysis for ITD-Mutant Exon-11 in *c-kit*

**Figure 4:**
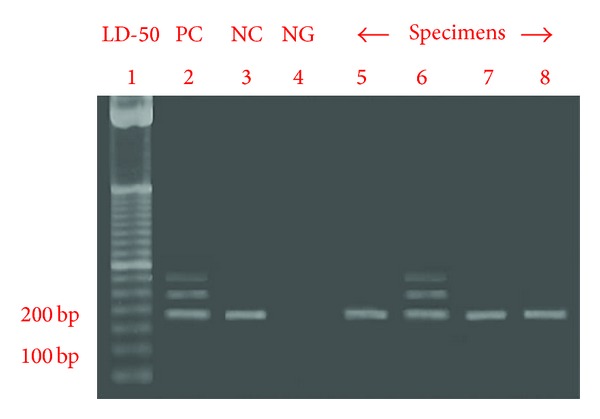
The PCR product of mutant specimen consisted of 3 subsidiary DNA bands (lane 6). The first band was a 191 bp DNA of normal mast cell gene allele, the middle band represented a 250 bp mutated DNA of exon-11, and the most upper band was a 300 bp heterogeneous DNA generating from a combination between normal allele and mutated DNA in MCT cells.

In this study, the PCR results resembled to those reported in tissue-based studies. The amplicon of mutant exon-11 was composed of 3 subsidiary DNA bands depending on its own product size. The first lowest band was 191 bp DNA of normal alleles, the middle band represented 250 bp mutant DNA of exon-11, and the upper band was 300 bp heteroduplex DNA arising from the combination between normal and mutant DNA alleles, as shown in Figure 4. From this study, the mutation of exon-11 in *c-kit* was detected only in high-grade MCT but not in low grade. In the overall aspect, there were 22 out of 30 FNA-MCT specimens (73.33%) possessed 191 bp of normal exon-11 in* c-kit* alleles; in a meantime, 26.67% (8/30) had mutant exon-11. This frequency was near to the distribution of this mutation formerly explained in a tissue-based study [13]. In addition, the data also suggested that FNA-MCT cells could be a source of cells to be utilized in mutational analysis of exon-11 in *c-kit*.

## 4. Discussion

MCT is a life-threatening skin tumor found in dogs [3]. The incidence is varied from 7 to 21% of all canine cutaneous tumors. The exact tumorigenesis of MCT is not well established yet; however, various *c-kit* mutations have been reported to be a driving force for MCT tumorogenesis [6, 10, 12–14, 16–18]. The most common mutation of *c-kit* reported in canine MCT is the ITD-mutation of exon-11 [19]. This mutation leading to an abnormal formation of KIT at the juxtamembrane domain and this abnormality gives rise to the autophosphorylation of KIT without any stem cell factor binding. The autophosphorylation causes uncontrollable proliferation and decreased apoptosis to mast cells resulting in tumor formation.

According to this study, the average age of studied MCT dogs was 9.6 years. There was no gender predilection seen in the study. The most prevalent breed was mixed breed (data not shown). Further, we have demonstrated that FNA-MCT cells provided a good source of neoplastic cells to be used to assess the biology of MCT cells, as shown in CD117-ICC and mutation evaluation by PCR.

By CD117-ICC, we have observed that the perimembrane pattern was predominantly present on the cell membranes of investigated FNA-MCT cells. It was only expressed in low-grade and not in a high-grade MCT. Perhaps, the cells in this grade were well differentiated like in mature mast cells. However, it is unclear why normal skin mast cells in the tissue-based study from Morini's group also expressed the diffuse pattern, albeit they were mature mast cells as well. Perhaps, it is possible that the diffuse pattern in those cells was caused by increased translations of *c-kit* under such circumstances, instead of an aberrant expression or transportation. Although, we did not see this pattern in our FNA-MCT cells from low-grade specimens. However, we believe that both perimembrane and diffusive patterns may strongly reflect an alteration in biological behavior of mast cells instead of indicating a histopathological grade, as observed in case of MCT.

Interestingly, the paranuclear pattern was found in our studied cells from both MCT grades and the result was harmonic to the study results from Morini, Preziosi, and Gil da Costa groups. This pattern was absent from the tissue-based normal mast cells, as shown up in the result from Morini's work. Perhaps the consequence has suggested that this pattern may be an indicative parameter for MCT formation particularly suggesting the transition phase from benign (low grade) to malignant (high grade) phase in MCT cells. Homologous to our study, this staining pattern was highly increased in the cells from high-grade MCT which had tremendously arbitrary biological behaviors. Therefore, the paranuclear pattern is maybe applied as a prognostic marker in the near future.

Based on PCR analysis, the result also suggested that stochastic fine-needle saspiration could provide enough number of FNA-MCT cells used for PCR detection of mutant exon-11. The PCR products usually consisted of 191 bp, 250 bp, and 300 bp subsidiary bands which were similar to the others reported in previous tissue-based studies. In almost mutant cases, the mutation was present in the cells which had the paranuclear staining pattern. Only two high-grade specimens with the diffuse pattern had mutant exon-11 in this study. Nonetheless, the mutant exon-11 was not reported in our low-grade specimens. Taken together, the study consequence has emphasized that the paranuclear pattern may be significant for MCT prognosis. In addition, the result also supported our hypothesis that there was an increased opportunity to observe a higher CD117-ICC staining pattern and exon-11 mutation in high-grade MCT; even these two parameters may not precisely indicate to a histopathological grade. This trend was also noted in a previous study by Webster et al. [13].

**Figure 5:**
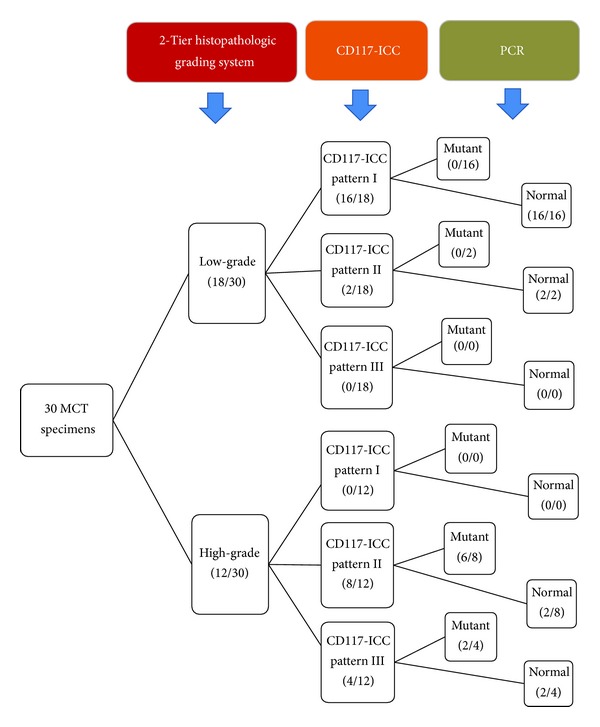
This drawing diagram exhibits the overall interrelations among histopathologic grading, CD117-ICC staining patterns, and PCR analysis of exon-11 mutation in *c-kit*. The consequence also provides the distribution of cases in each parameter.

The big picture of the probable interconnection of CD117-ICC staining patterns and ITD-mutant exon-11 to the 2-tier grading system can be summated in Figure 5.

In conclusion, the outcome in this study has loomed to the clue of the interconnections among these three parameters as shown in Figure 5. Basically, Patnaik's histopathologic grading system is a convenient tool for diagnosing MCT; however, the novel 2-tier grading system can enhance our ability to diagnose MCT more precisely than the first one. Moreover, this system also allows us to interpret our study results based on FNA-MCT cells easily. FNA is a clinically practical method to provide a quick assessment for MCT diagnosis. Likewise, this study has substantially shown the advanced benefit of FNA-MCT cell usage to investigate ITD-mutant exon-11 which might correlate to an abnormal CD117 localization. Thus, the result has implied that FNA-MCT cells themselves could give a deeply valuable data for MCT diagnosis and prognosis. Combined together, we believe that any novel diagnostic tool based on FNA-MCT cells will augment veterinary pathologists to make a quick assessment and prognosis for MCT in the future. For instance, in case of Toceranib administration, MCT dogs which have mutant exon-11 are usually responsive to this RTK-antagonist than nonmutated patients. Thus, any protocols which could detect mutant exon-11 swiftly must be excellent for mutant MCT patients. However, to the best of our knowledge, there are no alternative methods available for detecting the mutation except biopsy, so far. Also, this tool is so time-consuming that eventually it will be too late for MCT dogs to take the medicine. Thus, a further development of FNA-MCT-based tools is so essential, at least in this case.

Ultimately, we recommend that any future tools for MCT diagnosis and prognosis using FNA-MCT cells must be performed carefully with a large population using well-established protocols, before this method will be validly applied for MCT grading and prognosis [3, 20]. Particularly, the authentic correlation between any diagnostic parameters to histopathologic grades must be clarified.
